# Global Research Landscape and Thematic Evolution of Fungi-Derived Antimicrobials Against Methicillin-Resistant *Staphylococcus aureus* (MRSA): A Scientometric Analysis

**DOI:** 10.3390/biology15120967

**Published:** 2026-06-19

**Authors:** Christian Joseph N. Ong, Jamil Allen G. Fortaleza, Edison D. Ramos, Kevin Smith P. Cabuhat, Jowi Tsidkenu Pili Cruz, Amelda C. Libres, Joel G. Matamis, Jose Edwardo Mamaat, Carlos S. de Leon, Jose Jurel M. Nuevo

**Affiliations:** 1 Department of Biology, College of Science, De La Salle University, Manila 1004, Philippines; christian_joseph_ong@dlsu.edu.ph (C.J.N.O.); jowi.cruz@dlsu.edu.ph (J.T.P.C.); 2 National University, Manila 1008, Philippines; edramos@national-u.edu.ph; 3 Basic Education Department, La Consolacion University Philippines, Malolos 3000, Philippines; kevin_smith_cabuhat@dlsu.edu.ph; 4 College of Medical Laboratory Science, Liceo de Cagayan University, Cagayan deOro City 9000, Philippines; alibres@liceo.edu.ph; 5 School of Medical Laboratory Sciences, St. Dominic College of Asia, Cavite 4102, Philippines; jgmatamis@sdca.edu.ph; 6 Department of Medical Technology, Far Eastern University, Manila 1015, Philippines; jemamaat@feu.edu.ph; 7 Department of Medical Technology, Dr. Carlos S. Lanting College, Quezon City 1116, Philippines; 8 College of Medical Laboratory Science, Our Lady of Fatima University, Valenzuela City 1440, Philippines; jmnuevo@fatima.edu.ph

**Keywords:** fungi-derived antimicrobials, bioactive compounds, bibliometric analysis, natural products, translational microbiology

## Abstract

Methicillin-resistant *Staphylococcus aureus* (MRSA) remains a significant multidrug-resistant pathogen, frequently associated with persistent infections and biofilm formation, underscoring the urgent need for alternative antimicrobial strategies. Bioactive compounds derived from fungi have attracted considerable attention due to their structural diversity and demonstrated antibacterial activity against MRSA. This study employed a scientometric approach to assess global research trends, thematic evolution, and collaborative networks concerning fungi-derived anti-MRSA compounds. Bibliographic data were collected from the Scopus database, and a total of 1666 English-language articles and reviews published up to 2025 were analyzed using Bibliometrix/Biblioshiny and VOSviewer. The findings indicate a marked increase in research output after 2010, reflecting heightened scientific interest in fungal natural products for MRSA management. China and the United States emerged as leading contributors in terms of publication volume and international collaboration. Thematic analysis revealed a shift from broad antimicrobial screening to more specialized investigations, including antibiofilm activity, secondary metabolites, endophytic fungi, molecular docking, and antimicrobial resistance. Nonetheless, several challenges persist, such as insufficient mechanistic validation, limited toxicity and pharmacokinetic assessments, and a lack of clinically relevant *in vivo* studies. Overall, the field is increasingly multidisciplinary, integrating microbiology, natural product chemistry, and computational methodologies to advance the discovery of anti-MRSA agents.

## Simple Summary

Analysis of 1666 publications revealed a significant increase in scientific output after 2010, underscoring the growing significance of fungal natural products as alternative antimicrobial agents against MRSA. Scientometric evidence indicates a thematic shift from broad antimicrobial screening to more mechanistic and translational research areas, including antibiofilm activity, molecular docking, endophytic fungi, biosynthesis, and antimicrobial resistance. These developments illustrate the increasing integration of microbiology, natural product chemistry, and computational methods in anti-MRSA drug discovery. Overall, these findings offer a strategic framework for future research and facilitate the translation of promising fungal-derived bioactive compounds into clinically relevant interventions against MRSA and other multidrug-resistant bacterial pathogens.

## 1. Introduction

Methicillin-resistant *Staphylococcus aureus* (MRSA) is recognized as one of the most clinically significant multidrug-resistant pathogens globally, posing substantial challenges to healthcare systems due to its high morbidity and mortality, prolonged hospitalization, and rising treatment costs [1,2]. The rapid emergence and spread of MRSA strains resistant to multiple antibiotic classes, including β-lactams, glycopeptides, and, in some cases, vancomycin, have significantly restricted therapeutic options for both community-acquired and hospital-associated infections [3,4]. Additionally, MRSA’s capacity to form biofilms on medical devices and host tissues complicates infection management by promoting bacterial persistence, immune evasion, and tolerance to antimicrobial agents. As a result, the World Health Organization has designated MRSA as a priority pathogen, emphasizing the urgent need for novel antibacterial therapeutics and alternative antimicrobial strategies [5].

Amid the escalating antimicrobial resistance crisis, fungi have emerged as a significant source of structurally diverse bioactive metabolites with potent antibacterial properties. Endophytic fungi and other fungal taxa synthesize a wide array of secondary metabolites, including alkaloids, terpenoids, peptides, phenolics, and polyketides, many of which demonstrate promising anti-MRSA activity through various mechanisms [4,6,7]. These mechanisms encompass disruption of biofilm formation, inhibition of bacterial cell wall biosynthesis, interference with nucleic acid replication, and modulation of quorum-sensing (QS) pathways. Several fungal-derived compounds, such as gliotoxin and biosynthesized silver nanoparticles, have exhibited substantial bactericidal and antibiofilm activities against MRSA *in vitro* [8,9]. These findings underscore the potential of fungi-derived antimicrobials as alternative or complementary therapeutic agents for combating resistant bacterial pathogens.

Despite these advances, the translational development of fungi-derived antimicrobials is constrained by several scientific and technological challenges [4,6,10]. Most current investigations are limited to *in vitro* studies, while mechanistic elucidation, *in vivo* validation, pharmacokinetic characterization, toxicity profiling, and large-scale production have not been sufficiently addressed [4,6]. Furthermore, research in this field is fragmented across disciplines, geographical regions, and fungal taxa, which impedes the consolidation of knowledge required for therapeutic development. Although studies on fungal bioactive compounds against MRSA have increased in recent years, the broader scientific landscape, collaborative structures, thematic evolution, and emerging research directions remain insufficiently characterized.

Scientometric analysis provides a quantitative method for characterizing, assessing, and tracking bibliographical data (meta-data), forecasting research trends, and summarizing investigative progress [11,12]. Accordingly, this study utilizes a scientometric approach to examine the global research landscape of fungi-derived antimicrobials targeting MRSA. The specific objectives are to: (1) characterize temporal publication trends and growth dynamics; (2) identify the most productive countries, influential journals, and institutions; (3) map institutional and international collaboration networks; (4) analyze keyword co-occurrence and trending topics to identify dominant and emerging research themes; and (5) examine thematic mapping and thematic evolution, including factorial analysis to reveal conceptual developments, research frontiers, and future directions.

## 2. Materials and Methods

### 2.1. Study Design

The study employed scientometric approaches to characterize the structural evolution of research on fungi-derived anti-MRSA agents. Scientometrics enables the quantitative assessment of scientific productivity, collaborative networks, and thematic trajectories through analysis of bibliographic metadata, co-authorship linkages, and keyword co-occurrence patterns. This approach offers a systematic means to trace the growth, diversification, and emerging directions of a research field.

### 2.2. Data Source

Scopus was selected as the primary data source for this study because of its extensive coverage of fungal biology and applied microbiology journals, robust citation indexing, and established application in scientometric research. Compared with Clarivate’s Web of Science (WoS), Scopus provides broader coverage of global and applied microbiology and fungal biology journals, making it particularly appropriate for mapping the diverse research landscape of fungi-derived antimicrobials targeting MRSA. Utilizing Scopus as the sole bibliographic source ensures high metadata uniformity and substantially reduces the risk of duplicate records within the longitudinal dataset. Although retrieving data from multiple databases can slightly increase coverage, it often introduces significant inconsistencies in citation indexing and metadata, particularly in author naming conventions, institutional affiliations, and keyword standardization. These discrepancies necessitate extensive post hoc reconciliation and may introduce systematic bias due to imperfect record matching or fragmented citation counts across platforms. Restricting data retrieval to a single, comprehensive database maintains internal consistency and standardization of bibliometric fields, such as authorship, institutional and country affiliations, and citation counts, which are essential for reliable network construction and reproducible citation-based indicators. This methodology is consistent with established scientometric practices, where the primary aim is structural mapping and relational analysis rather than the exhaustive synthesis characteristic of systematic reviews. Additionally, Scopus enables exporting detailed metadata, including subject classifications and CiteScore indicators, which help contextualize source influence. The extracted metadata comprised publication year, authorship, institutional affiliations, journal sources, keywords, abstracts, subject categories, and citation counts. However, reliance on a single database may underrepresent locally published or non-indexed research from lower-income regions. Therefore, observed disparities in research output should be interpreted as differences in indexed scholarly visibility rather than as absolute measures of research activity.

### 2.3. Search Strategy and Data Extraction

**Figure 1:**
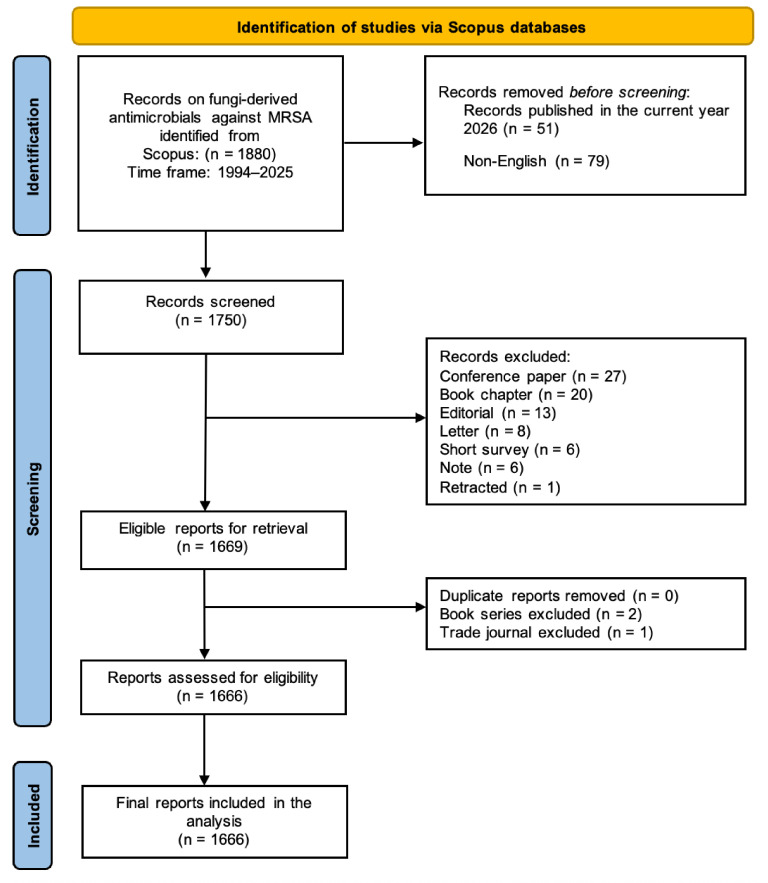
PRISMA flow diagram of the scientometric study on fungi-derived antimicrobials against MRSA.

A structured search strategy was developed to maximize retrieval sensitivity while maintaining clinical specificity and global relevance. Data retrieval was conducted in Scopus on 19 May 2026, using the TITLE-ABS-KEY fields to capture publications in which validated keywords related to fungi-derived antimicrobials against methicillin-resistant *Staphylococcus aureus* (MRSA) appeared in article titles, abstracts, or author/index keywords. To improve transparency and reproducibility in bibliographic retrieval and screening, the study selection workflow was adapted from PRISMA-style reporting principles, including record identification, screening, eligibility assessment, and final inclusion of publications for scientometric analysis (Figure 1).

The query incorporated a comprehensive set of taxonomic, pharmacological, and clinical descriptors to maximize retrieval of relevant literature on fungi-derived antimicrobials against methicillin-resistant *Staphylococcus aureus* (MRSA). Broad fungal-related terms such as “mushroom,” “macrofungi,” “basidiomycota,” “ascomycota,” “medicinal mushroom,” “edible mushroom,” “higher fungi,” and “fungal” were included to capture studies involving diverse fungal taxa and both medicinal and edible macrofungi. Antimicrobial-related keywords, including “antimicrobial,” “antibacterial,” “antibiotic,” “anti-biofilm,” “antibiofilm,” “anti-infective,” “bactericidal,” and “bacteriostatic,” were incorporated to encompass multiple antibacterial mechanisms and therapeutic applications. To specifically target research on resistant *Staphylococcus aureus*, the query included “MRSA,” “methicillin-resistant Staphylococcus aureus,” “drug-resistant Staphylococcus aureus,” and “multidrug-resistant Staphylococcus aureus.”

To enhance data stability and citation accuracy, publications indexed in 2026 were excluded due to incomplete data collection and variability in indexing at the time. The inclusion criteria were limited to peer-reviewed original research and review articles published in English-language journals to ensure consistency in bibliometric measures and analysis. Non-research documents, such as editorials, letters, conference proceedings, notes, and book chapters, were excluded to maintain homogeneity across productivity and citation analyses. The search strategy focused on including fungal studies related to antimicrobials, fungal taxonomy, and MRSA to facilitate ecosystem-level mapping of the global research landscape. Selected records were exported in CSV format with comprehensive bibliographic metadata for preprocessing and analysis. Journal-level CiteScore metrics were obtained directly from Scopus to provide context for source influence within the research ecosystem.

The Scopus advanced search query was designed to capture scholarly articles on fungi-derived antimicrobials against MRSA and was applied to article titles and abstracts. The full search string used in this study is presented below:

TITLE-ABS-KEY ((mushroom* OR macrofung* OR basidiomyc* OR ascomyc* OR “medicinal mushroom*” OR “edible mushroom*” OR “higher fungi” OR fungal) AND (antimicrobial* OR antibacterial* OR antibiotic* OR “anti-biofilm” OR antibiofilm OR “anti-infective*” OR bactericidal OR bacteriostatic) AND (MRSA OR “methicillin-resistant Staphylococcus aureus” OR “drug-resistant Staphylococcus aureus” OR “multidrug-resistant Staphylococcus aureus”)) AND (EXCLUDE (PUBYEAR, “2026”)) AND (LIMIT-TO (DOCTYPE, “ar”) OR LIMIT-TO (DOCTYPE, “re”)) AND (LIMIT-TO (LANGUAGE, “English”)) AND (LIMIT-TO (SRCTYPE, “j”)).

### 2.4. Data Cleaning and Harmonization

To ensure data accuracy, consistency, and analytical reliability, a comprehensive manual data-cleaning procedure was performed on the extracted dataset prior to bibliometric analysis. First, duplicate records were identified and removed, and author names, institutional affiliations, and country information were standardized to minimize inconsistencies caused by variations in indexing formats. Manual screening of titles and abstracts was subsequently conducted to exclude irrelevant publications not directly related to fungi-derived antimicrobial research against methicillin-resistant Staphylococcus aureus (MRSA), even when they contained overlapping search terms. This step helped reduce database noise and improve dataset specificity.

Keyword harmonization was performed using a manually curated thesaurus file (.txt) to consolidate synonymous terms, spelling variants, abbreviations, and singular–plural forms into unified descriptors. For example, “methicillin resistant Staphylococcus aureus,” “MRSA,” and “methicillin-resistant S. aureus” were merged under the term “MRSA”; “antibacterial activity,” “antimicrobial activity,” and “antimicrobial effect” were standardized as “antimicrobial activity”; and “endophytic fungi” and “endophytic fungus” were unified as “endophytic fungi.” Similar harmonization was applied to taxonomic names, bioactive compounds, and methodological terms to ensure consistency in network construction. Following data cleaning, minimum thresholds were applied for network analyses (≥5 keyword occurrences and ≥10 institutional publications) to reduce noise, improve visualization clarity, and enhance the interpretability of bibliometric networks.

### 2.5. Data Analysis

**Figure 2:**
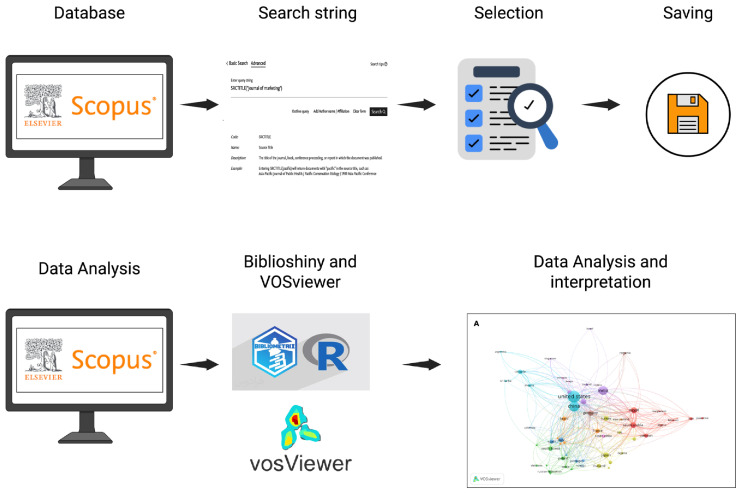
Schematic workflow of the scientometric analysis conducted in this study, illustrating the processes of database retrieval from Scopus, search strategy formulation, study selection and data exportation, followed by enrichment and science mapping analyses using Bibliometrix/Biblioshiny (R package) and VOSviewer for visualization.

This scientometric study conducted science mapping and performance analysis using the retrieved dataset (.csv) through enrichment tools, such as Bibliometrix/Biblioshiny (R package) [13] and VOSviewer version 1.6.20. (Universiteit Leiden and CWTS Meaningful Metrics). Bibliometrix/Biblioshiny (R) and VOSviewer were both used for visualizations, such as network maps, thematic diagrams, and trend figures, and conceptual structure mapping through strategic thematic mapping, thematic evolution and multidimensional scaling (MDS). Publication and citation metrics, including annual publication counts, top journals, trend topics and leading institutions, were computed using Bibliometrix. Co-authorship and co-occurrence networks were mapped in VOSviewer to characterize inter-country and inter-institutional collaborations, as well as centrality structures of keywords, accordingly (Figure 2). Thematic evolution was assessed using keyword co-occurrence analysis of titles and abstracts, producing cluster maps and temporal overlays to identify dominant research areas and emerging fronts.

## 3. Results

### 3.1. Research Trend and Temporal Dynamics

**Figure 3:**
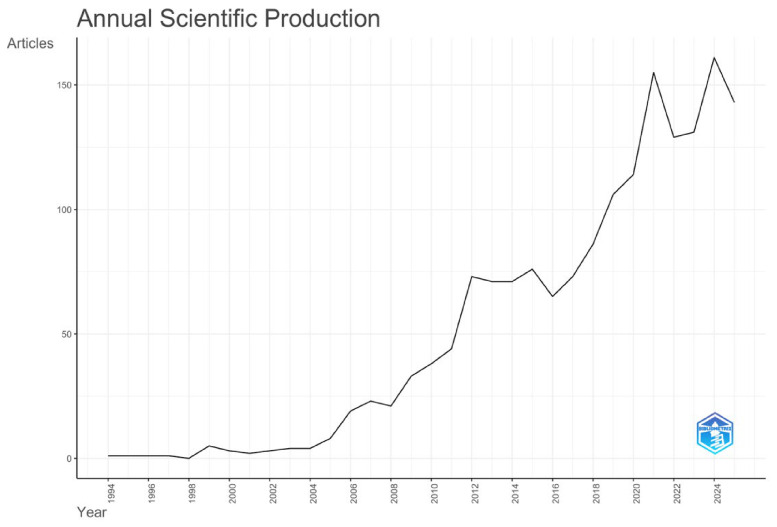
Trend analysis of global annual scientific publications on fungi-derived antimicrobials against MRSA research.

A total of 1666 documents, including research articles and reviews, were retrieved from the Scopus database and analyzed. The annual scientific output on fungi-derived antimicrobials against MRSA increased steadily from limited publications in the mid-1990s to a highly active research field by the 2020s. Publication output remained relatively low until the early 2000s, then grew gradually between 2005 and 2010. A more substantial increase was observed after 2011–2012, indicating growing scientific interest in fungal metabolites and natural-product-based anti-MRSA agents (Figure 3). From 2017 onward, publication activity increased rapidly, with annual outputs exceeding 100 articles by around 2019 and remaining consistently high through the early 2020s. Although minor fluctuations were observed after 2020, the overall trend suggests continued expansion in research on fungi-derived antimicrobials against MRSA, likely driven by growing concerns about antimicrobial resistance and the search for alternative antimicrobial compounds.

**Figure 4:**
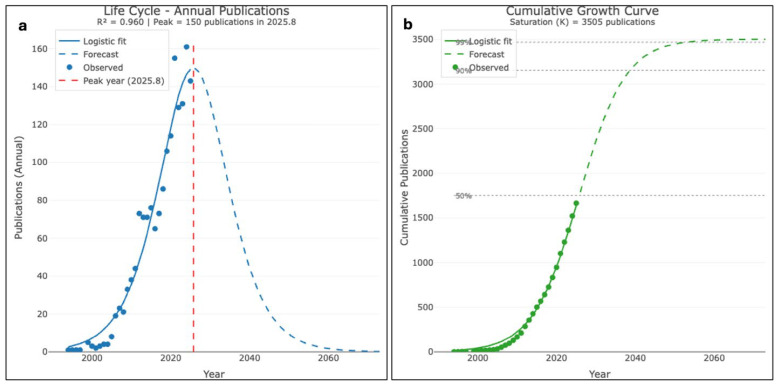
Life cycle of global annual scientific production (**a**) and the cumulative growth curve (**b**) of research on fungi-derived antimicrobials against MRSA.

Global research on fungi-derived antimicrobials against MRSA showed a strong upward trend, with the logistic growth model providing a good fit to the annual publication pattern (R^2^ = 0.960), as shown in Figure 4a. Publication output remained low before the 2000s, increased gradually during the early growth phase, and accelerated markedly after the 2010s, reaching a projected peak of approximately 150 publications around 2025 (Figure 4b). This trend reflects increasing scientific interest in fungal natural products as potential anti-MRSA agents.

The cumulative growth curve further suggests continuous expansion of the literature, with an estimated carrying capacity of approximately 3505 publications. The curve shows slow early accumulation, followed by rapid growth after the mid-2010s, and a projected gradual stabilization in the coming years. Overall, these growth patterns indicate that the field has evolved from an emerging research area into a more established, increasingly specialized domain, with future studies likely to focus on mechanistic characterization, validation, and preclinical translational investigations rather than on broad exploratory screening alone.

### 3.2. Geographical Distribution of Scientific Publications

**Figure 5:**
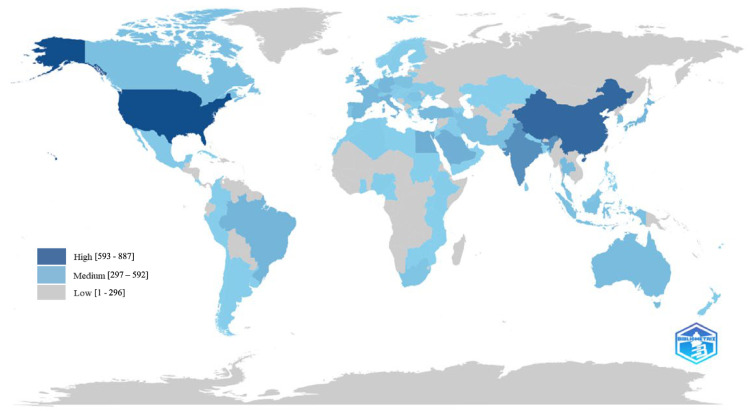
Country-specific scientific production of research on fungi-derived antimicrobials against MRSA.

The country-specific production map shows that research on fungi-derived antimicrobials against MRSA is globally distributed but unevenly concentrated across regions (Figure 5). China and the United States emerged as the leading contributors, reflecting strong research capacity in antimicrobial resistance, natural-product chemistry, fungal biotechnology, and antimicrobial discovery. Moderate research productivity was also observed across parts of Europe, South Asia, Southeast Asia, Latin America, and Oceania, indicating broader international participation in fungi-derived antimicrobial research. In contrast, many countries in Africa, Central Asia, and parts of Eastern Europe showed limited indexed scientific output. These disparities may be associated with differences in research funding, laboratory infrastructure, documentation of fungal biodiversity, antimicrobial screening capacity, and visibility in major bibliographic databases. Although research on fungi-derived anti-MRSA has become increasingly international, scientific productivity remains concentrated in highly resourced regions. Expanding collaboration with underrepresented countries may enhance fungal bioprospecting efforts and support the discovery of taxonomically and ecologically diverse antimicrobial compounds.

### 3.3. Scientific Co-Authorship and Collaboration Network

**Figure 6:**
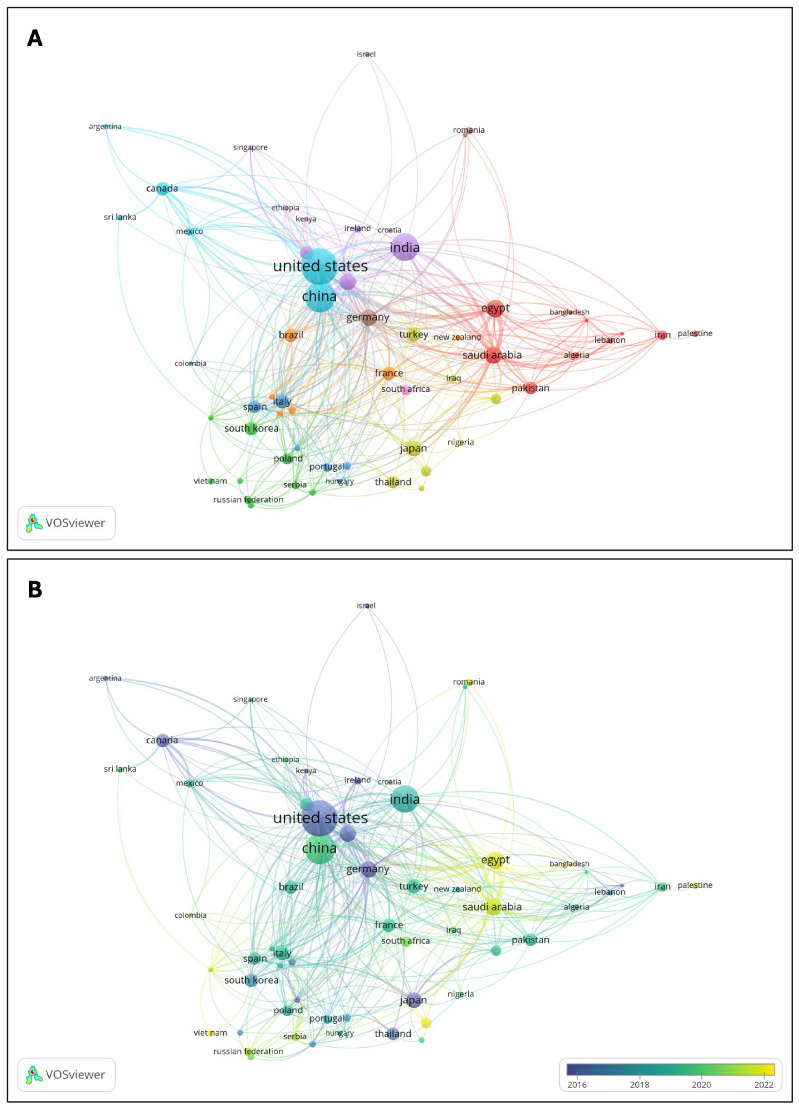
Co-authorship of research on fungi-derived antimicrobials against MRSA among countries and collaboration through (**A**) network analysis, and (**B**) overlay visualization.

The country co-authorship network shows that research on fungi-derived antimicrobials against MRSA is globally interconnected yet unevenly distributed across major scientific hubs. In the network map (Figure 6A), larger nodes such as China, the United States, India, and Iran indicate countries with higher publication and collaboration activity, while dense links among these countries suggest frequent international co-authorship. The clustering pattern further reveals several regional collaboration groups, with China and the United States serving as major collaborative centers that link Asian, Middle Eastern, European, and American research communities. Other countries, including Saudi Arabia, Egypt, Malaysia, Pakistan, South Korea, and Brazil, also emerged as important contributors, demonstrating increasing participation from Asia-Pacific and Global South research systems.

The overlay visualization (Figure 6B) suggests a temporal shift in collaboration patterns, with earlier research activity concentrated in established scientific hubs and more recent contributions emerging from countries such as Egypt, Saudi Arabia, Pakistan, Bangladesh, and parts of Southeast Asia. This trend indicates that research on fungi-derived anti-MRSA has gradually become more geographically diverse. The network and overlay visualizations collectively demonstrate strong international collaboration among major research-producing countries, particularly China, the United States, and India, as well as several emerging contributors from Asia and the Middle East.

### 3.4. Trend and Burning Topics

**Figure 7:**
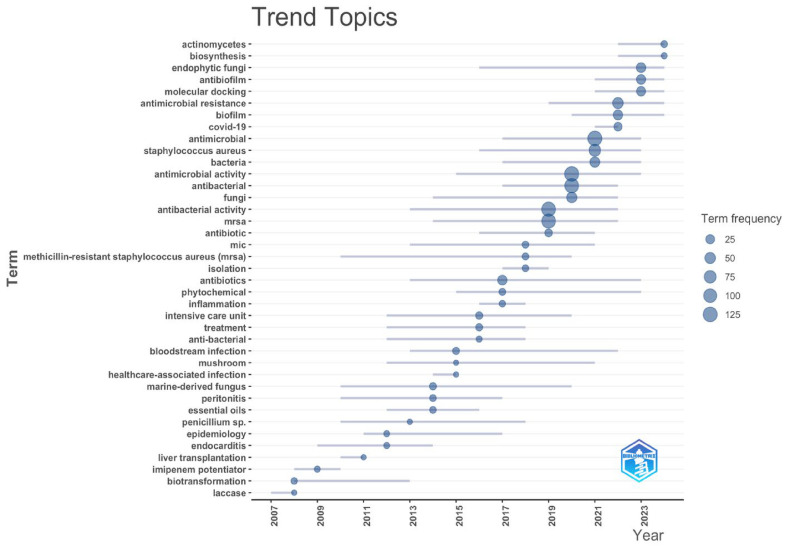
Topics studied over time in relation to fungi-derived antimicrobials against MRSA.

The trend-topic analysis shows a clear thematic transition from early, exploratory, source-oriented studies to more targeted antimicrobial and anti-MRSA investigations. Earlier terms such as “laccase,” “biotransformation,” “endocarditis,” “epidemiology,” “*Penicillium* sp.,” “essential oils,” and “marine-derived fungus” suggest that the field initially emphasized fungal metabolites, enzymatic transformation, clinical infection contexts, and natural-product discovery. By the mid-2010s, the literature increasingly incorporated terms such as “phytochemical,” “antibiotics,” “treatment,” “intensive care unit,” “bloodstream infection,” and “methicillin-resistant Staphylococcus aureus (MRSA),” indicating a shift toward clinical relevance and antimicrobial-resistance applications (Figure 7).

From 2019 onward, the field became increasingly focused on themes related to “antibacterial activity,” “antimicrobial activity,” “fungi,” “Staphylococcus aureus,” “antimicrobial resistance,” and “biofilm,” indicating continued emphasis on fungal-derived antimicrobial screening and anti-MRSA investigations. More recent terms such as “molecular docking,” “endophytic fungi,” “biosynthesis,” “actinomycetes,” and “antibiofilm” suggest a growing shift toward mechanistic and preclinical research approaches involving computational screening, fungal metabolite discovery, biosynthetic pathway analysis, and biofilm-targeted antimicrobial strategies.

**Figure 8:**
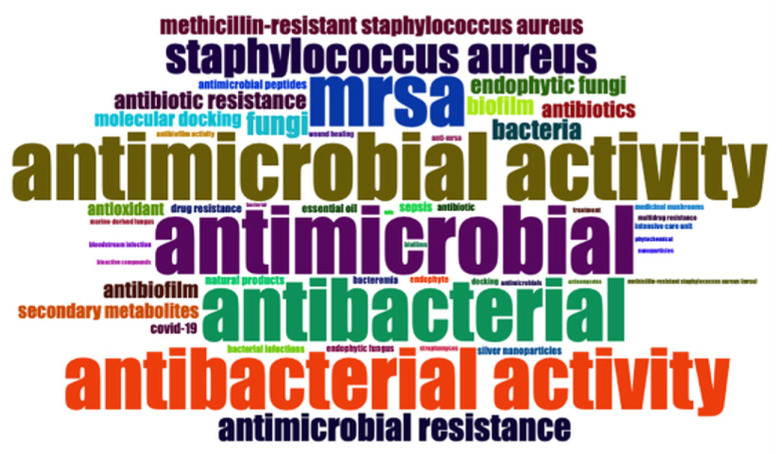
The word cloud visualizes the most frequently occurring terms in fungi-derived anti-MRSA research, highlighting themes in the field.

The word cloud further demonstrates that research on fungi-derived antimicrobials against MRSA is strongly centered on antibacterial screening and antimicrobial activity (Figure 8). Highly prominent terms such as “antimicrobial activity,” “antibacterial activity,” “antibacterial,” “antimicrobial,” and “MRSA” indicate that the field is primarily focused on evaluating the inhibitory potential of fungal-derived extracts, metabolites, and natural products against resistant *Staphylococcus aureus*. The frequent occurrence of terms such as “biofilm,” “antibiofilm,” “antimicrobial resistance,” and “antibiotic resistance” further suggests growing scientific attention to resistance-associated persistence mechanisms and biofilm-related infections, which are major contributors to MRSA pathogenicity and therapeutic failure.

In addition, the appearance of terms such as “molecular docking,” “endophytic fungi,” “secondary metabolites,” “natural products,” and “silver nanoparticles” reflects the growing integration of computational screening, fungal metabolite discovery, and nanotechnology-assisted antimicrobial approaches within the field. Collectively, the word cloud suggests that fungi-derived anti-MRSA research has gradually expanded beyond broad antimicrobial screening toward more mechanistic and preclinical investigations involving antibiofilm activity, resistance mitigation, and natural-product-based antimicrobial discovery.

The increasing prominence of molecular docking in recent years reflects the integration of computational approaches into natural product research. Molecular docking enables rapid prediction of compound–target interactions. It facilitates lead prioritization and reduces the time and cost associated with experimental screening. These advantages make it a valuable tool for accelerating antimicrobial drug discovery. The emergence of antibiofilm activity as a major research theme corresponds with growing recognition of the role of biofilms in MRSA persistence, chronic infections, and antimicrobial tolerance. Since biofilm-associated infections are often resistant to conventional antibiotic therapy, there is increasing interest in identifying fungal metabolites that can disrupt biofilm formation or enhance antibiotic susceptibility. The heightened focus on endophytic fungi is driven by their capacity to produce structurally diverse secondary metabolites with unique biological activities. As symbiotic microorganisms inhabiting plant tissues, endophytic fungi represent an underexplored reservoir of bioactive compounds. They have become a significant target for bioprospecting efforts to discover novel antimicrobial agents against multidrug-resistant pathogens such as MRSA.

### 3.5. Most Relevant Sources

Table 1 shows that publications on fungi-derived antimicrobials against MRSA are concentrated in journals specializing in natural products, microbiology, antibiotics, and molecular sciences. The Journal of Natural Products ranked first in output (46 publications), total citations (1991), and h-index (24), underscoring its central role as both a productive and influential venue. Frontiers in Microbiology and the Journal of Antibiotics followed with 38 and 36 publications, respectively, reflecting the field’s strong focus on microbiology and antimicrobial discovery. Although Molecules ranked fourth in productivity, it recorded the highest average citations per paper (52.17), suggesting that its contributions had a particularly strong citation impact.

The remaining journals further demonstrate the multidisciplinary nature of fungi-derived anti-MRSA research, spanning phytochemistry, marine natural products, pharmacology, microbiology, antibiotics, and molecular sciences. MDPI journals were particularly prominent, with Molecules, Antibiotics, Marine Drugs, and the International Journal of Molecular Sciences appearing among the top-publishing sources, highlighting the important role of broad-scope open-access journals in disseminating research on antimicrobial resistance and natural products. Among these journals, Marine Drugs recorded the highest CiteScore (10.1) and a strong citation profile, reflecting growing scientific interest in marine-derived fungi and marine natural products as potential sources of anti-MRSA compounds. Overall, the publication landscape suggests that the field remains strongly rooted in natural-product chemistry while increasingly expanding into microbiology, molecular biosciences, and antimicrobial-resistance research.

### 3.6. Most Leading Institutions

The institutional productivity analysis indicates that research on fungi-derived antimicrobials against MRSA is strongly led by Asian research systems, particularly China. The Chinese Academy of Sciences emerged as the most productive institution with 55 publications, followed by the University of Chinese Academy of Sciences with 29 publications. Other Chinese institutions, including the Ministry of Education of the People’s Republic of China and the Institute of Microbiology, Chinese Academy of Sciences, also appeared among the top contributors (Table 2). These findings reflect China’s strong institutional capacity in fungal natural-product discovery, antimicrobial screening, and MRSA-related research.

Beyond China, the institutional landscape demonstrates broader but uneven international participation. King Saud University ranked third with 28 publications, followed by CNRS in France, Al-Azhar University in Egypt, Prince of Songkla University and the Thailand National Center for Genetic Engineering and Biotechnology in Thailand, and the University of Mississippi in the United States. The presence of institutions from Saudi Arabia, Egypt, Thailand, France, and the United States highlights the contribution of both established research centers and emerging institutions from biodiverse regions. Overall, the findings suggest that research on fungi-derived anti-MRSA is shaped by strong Asian leadership, alongside growing participation from the Middle East, Southeast Asia, Europe, and North America.

### 3.7. Keyword Co-Occurrence Network Analysis

**Figure 9:**
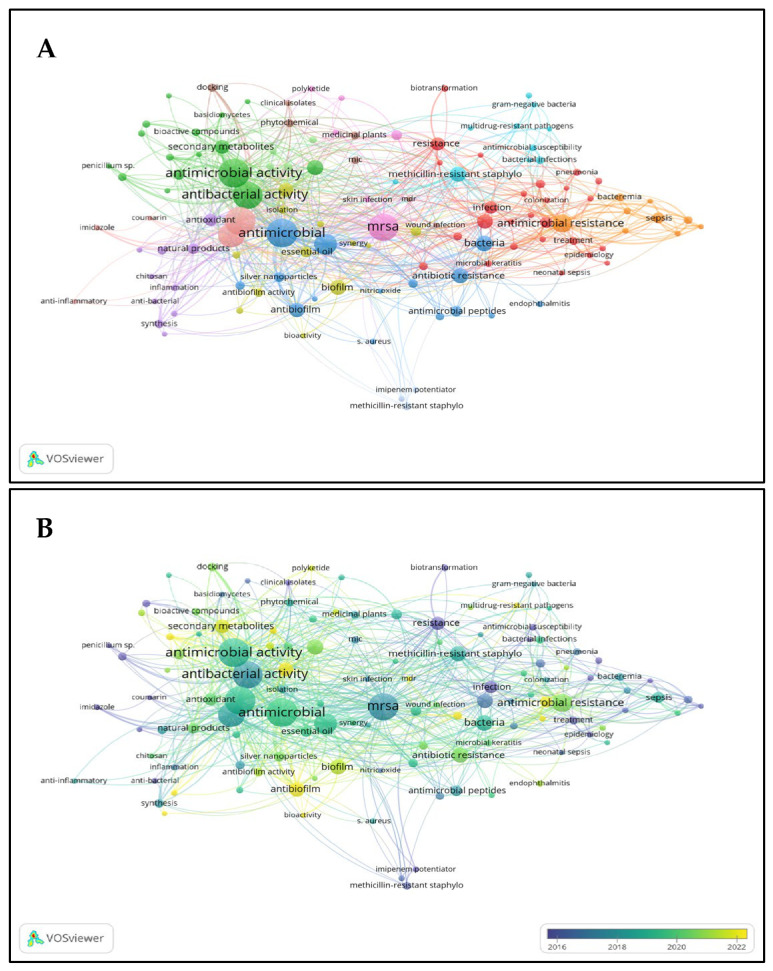
Keyword co-occurrence through (**A**) network visualization and (**B**) overlay visualization of fungi-derived antimicrobials against MRSA.

The keyword co-occurrence network shows that research on fungal-derived antimicrobials against MRSA is organized into several interconnected thematic clusters. In Figure 9A, the largest and most central terms—“antimicrobial activity,” “antibacterial activity,” “antimicrobial,” “MRSA,” “Staphylococcus aureus,” and “antimicrobial resistance”—indicate that the field is primarily focused on evaluating fungal-derived compounds or extracts for antibacterial efficacy against resistant staphylococcal pathogens. Closely linked terms such as “biofilm,” “antibiofilm,” “secondary metabolites,” “natural products,” “essential oils,” “molecular docking,” and “silver nanoparticles” suggest that the literature extends beyond growth inhibition to include mechanisms of action, biofilm suppression, phytochemical/natural-product discovery, and nanotechnology-assisted antimicrobial strategies.

The overlay visualization in Figure 9b indicates a temporal shift in the research landscape. Earlier studies emphasize broad antimicrobial screening, fungal metabolites, and natural-product sources, whereas more recent terms are associated with “antimicrobial resistance,” “biofilm,” “molecular docking,” “endophytic fungi,” “biosynthesis,” and “antibiotic resistance.” This progression suggests that the field has matured from descriptive screening of fungal extracts to a more mechanistic and translational domain focused on mitigating resistance, antibiofilm activity, computational prioritization of bioactive compounds, and the discovery of novel metabolites from endophytic or specialized fungal sources.

### 3.8. Conceptual Structure Mapping

**Figure 10:**
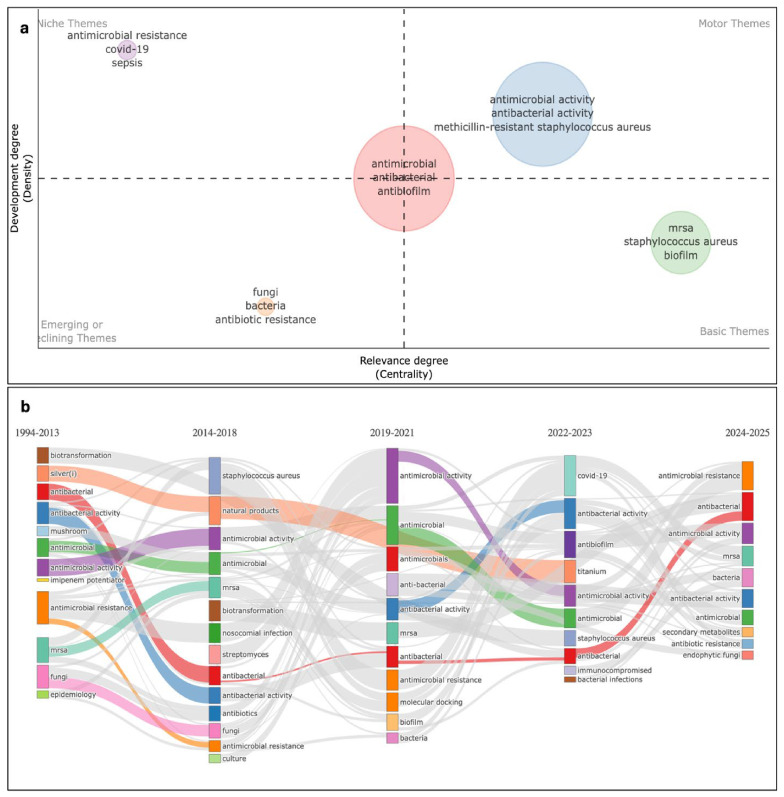
Strategic thematic map (**a**) and thematic evolution (**b**) of research on fungi-derived antimicrobials against MRSA.

The strategic thematic map indicates that research on fungi-derived antimicrobials against MRSA is primarily focused on antimicrobial screening and anti-MRSA activity (Figure 10a). The motor theme cluster, comprising “antimicrobial activity,” “antibacterial activity,” and “methicillin-resistant *Staphylococcus aureus,*” represents the most developed and influential research area, highlighting the continued focus on identifying fungal extracts, metabolites, and natural products with inhibitory effects against MRSA. In contrast, the cluster containing “MRSA,” “Staphylococcus aureus,” and “biofilm” appears as a basic theme, suggesting that although biofilm-related investigations are highly relevant, they remain comparatively less developed and may still require stronger mechanistic characterization and standardized antibiofilm approaches.

The niche and emerging-or-declining quadrants further provide insight into evolving research priorities within the field. Terms such as “antimicrobial resistance,” “COVID-19,” and “sepsis” appeared as niche themes, indicating specialized but less integrated areas of investigation within fungi-derived antimicrobial research. Meanwhile, broader terms, including “fungi,” “bacteria,” and “antibiotic resistance,” occupied the emerging or declining quadrant, possibly reflecting either early-stage development or declining thematic centrality as the field becomes more specialized. The centrally positioned cluster involving “antimicrobial,” “antibacterial,” and “antibiofilm” appears to function as a bridging theme connecting general antimicrobial discovery with studies focused on biofilm inhibition and virulence-related mechanisms. Overall, the thematic structure suggests a gradual transition from broad antibacterial screening toward more mechanism-oriented and application-focused anti-MRSA investigations.

The thematic evolution map further demonstrates that the field progressed from broad exploratory themes between 1994 and 2013, including “fungi,” “mushroom,” “biotransformation,” “MRSA,” and “antimicrobial activity,” toward more focused pathogen- and natural-product-oriented themes during 2014–2018 (Figure 10b). Between 2019 and 2023, the emergence of terms such as “molecular docking,” “biofilm,” “antimicrobial resistance,” “antibiofilm,” and “antibacterial activity” indicates increasing integration of computational screening, mechanistic analysis, and resistance-focused research approaches. More recent themes observed during 2024–2025, including “secondary metabolites,” “endophytic fungi,” and “antibiotic resistance,” further suggest growing interest in fungal metabolite discovery and preclinical anti-MRSA investigations. Future studies may benefit from integrating metabolomics, genome mining, standardized MRSA biofilm models, toxicity assessment, and *in vivo* validation to strengthen the translational relevance of fungi-derived antimicrobial candidates.

**Figure 11:**
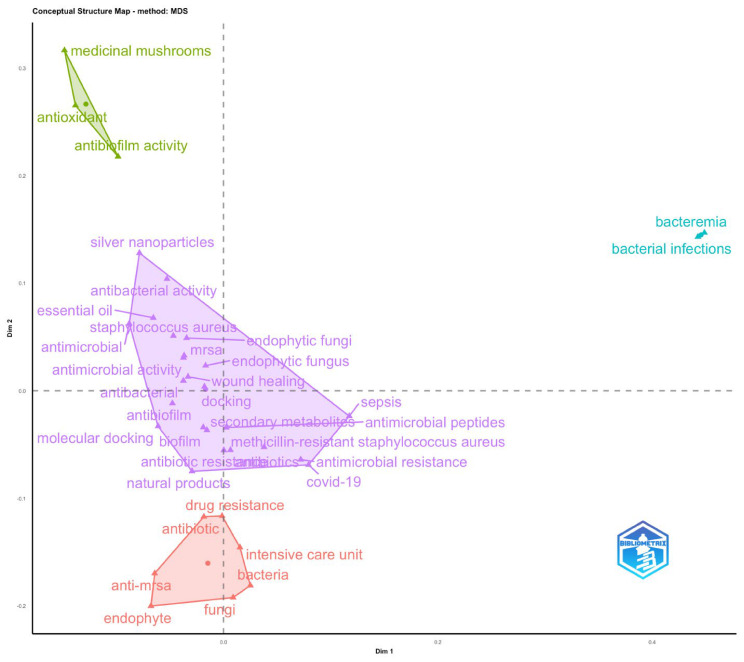
Factorial analysis through four-clustered multidimensional scaling (MDS) of research on fungi-derived antimicrobials against MRSA.

The factorial analysis based on multidimensional scaling (MDS) demonstrates a conceptually diverse but thematically interconnected research landscape for fungi-derived antimicrobials against MRSA (Figure 11). The largest and most central cluster is composed of terms such as “antimicrobial activity,” “antibacterial activity,” “antibiofilm,” “biofilm,” “MRSA,” “*Staphylococcus aureus*,” “endophytic fungi,” “secondary metabolites,” “molecular docking,” and “natural products.” The central positioning of these terms indicates that they represent the intellectual core of the field, linking fungal natural-product discovery with antibacterial screening, antibiofilm investigations, and computational approaches for candidate compound prioritization. This pattern suggests that most studies remain focused on activity-based screening while increasingly incorporating mechanistic and preclinical research approaches related to biofilm inhibition, antimicrobial resistance, and metabolite discovery.

The smaller clusters represent more specialized research subdomains. The upper-left cluster, containing terms such as “medicinal mushrooms,” “antioxidant,” and “antibiofilm activity,” reflects a bioactivity-oriented research area investigating mushrooms as sources of multifunctional compounds with antimicrobial and antioxidant properties. Meanwhile, the lower-left cluster, including “drug resistance,” “antibiotic,” “intensive care unit,” “bacteria,” “fungi,” “endophyte,” and “anti-MRSA,” appears to connect fungal-derived compounds with clinical antimicrobial resistance contexts and hospital-associated infections. In contrast, the smaller right-sided cluster, comprising “bacteremia” and “bacterial infections,” remains relatively separate from the central thematic structure, suggesting that clinically relevant infection outcomes remain weakly integrated with fungal-derived antimicrobial discovery.

Overall, the MDS structure indicates that the field has achieved conceptual consolidation around anti-MRSA activity, biofilm control, endophytic fungi, and secondary metabolites, yet still shows limited integration between discovery-stage investigations and clinical applications. The separation of clinical terms such as “bacteremia,” “sepsis,” and “intensive care unit” from the main natural-product cluster highlights an existing translational gap between *in vitro* antimicrobial screening and clinically relevant infection models. Future investigations may therefore benefit from greater emphasis on standardized MRSA and biofilm assays, compound isolation and structural validation, omics-guided metabolite discovery, toxicity assessment, pharmacological characterization, and *in vivo* infection models to improve the translational relevance of fungi-derived anti-MRSA candidates.

## 4. Discussion

### 4.1. Global Research Landscape and Thematic Evolution

The scientometric findings demonstrate that research on fungi-derived antimicrobials against methicillin-resistant *Staphylococcus aureus* (MRSA) has evolved from a relatively niche natural-products discipline into a rapidly expanding and globally interconnected research field. The marked increase in publication output after the 2010s reflects growing scientific urgency to identify alternative therapeutic strategies amid the escalating antimicrobial resistance (AMR) crisis. This expansion parallels growing concerns about the declining efficacy of conventional antibiotics against multidrug-resistant *S. aureus*, particularly in healthcare-associated and biofilm-mediated infections that are associated with substantial morbidity and mortality worldwide [14,15]. The intensified scientific interest in fungal metabolites and mushroom-derived compounds likely stems from the recognition that fungi constitute a rich reservoir of structurally diverse secondary metabolites with antibacterial, antibiofilm, and anti-virulence properties [16]. The observed growth trajectory, therefore, reflects broader efforts to diversify antimicrobial discovery pipelines beyond conventional synthetic antibiotics.

The geographical and institutional distribution patterns indicate that research on fungi-derived anti-MRSA agents remains concentrated in scientifically advanced and biodiversity-rich regions, particularly China, the United States, Saudi Arabia, and several Southeast Asian countries. China emerged as the dominant contributor in both productivity and collaborative influence, likely reflecting sustained national investments in biotechnology, natural-product chemistry, metabolomics, and antimicrobial drug discovery. Previous studies have shown that China has substantially expanded its scientific productivity, patent generation, and international research influence over recent decades [17,18]. Similarly, the strong contribution of the United States may reflect its advanced biotechnology infrastructure, translational microbiology expertise, and integration of computational biology with antimicrobial discovery [19,20]. The increasing participation of Southeast Asian and Middle Eastern institutions may additionally be associated with high regional biodiversity and growing awareness of AMR-related healthcare burdens. Tropical ecosystems, marine environments, and endophytic fungal niches remain important reservoirs of chemically unique antimicrobial metabolites with potential anti-MRSA applications [21,22,23].

Despite the increasing globalization of the field, substantial disparities remain evident across the international research landscape. Africa, Central Asia, and many low-income countries remain weakly represented despite possessing ecologically rich environments with potentially valuable fungal diversity. Similar inequities have been documented across multiple scientific disciplines and are frequently associated with limited research funding, inadequate laboratory infrastructure, restricted access to advanced sequencing and metabolomic technologies, and publication inequalities favoring highly resourced countries [24,25,26]. In addition, researchers from low-resource settings often face barriers such as limited international collaboration, reduced access to advanced analytical platforms, and lower visibility in highly cited journals [27,28]. Consequently, the current scientometric landscape may reflect disparities in the visibility of indexed research rather than the true distribution of fungal biodiversity or scientific potential. Strengthening equitable international collaboration, biodiversity partnerships, and open-access metabolomic resources may therefore improve globally representative fungal bioprospecting and expand the discovery pipeline for novel anti-MRSA compounds [29,30].

### 4.2. Thematic Evolution and Emerging Research Directions

The thematic evolution and keyword co-occurrence analyses indicate substantial conceptual maturation within fungi-derived anti-MRSA research. Earlier investigations primarily emphasized crude extract screening and broad-spectrum antibacterial testing, reflecting the exploratory nature of early fungal natural-product research [31,32]. During this phase, fungal metabolites such as alkaloids, terpenoids, peptides, and polyketides were broadly investigated for antimicrobial potential against diverse pathogens [33,34]. These early studies established the foundational evidence that fungi produce chemically diverse secondary metabolites with potential therapeutic applications, thereby supporting subsequent expansion of fungal antimicrobial research.

More recent literature increasingly incorporates themes related to molecular docking, endophytic fungi, biosynthetic pathways, antibiofilm activity, and antimicrobial resistance mechanisms, suggesting a progressive shift toward mechanism-oriented and translational research frameworks. This transition is scientifically important because MRSA pathogenicity is strongly associated with biofilm formation, quorum sensing, toxin production, and adaptive resistance phenotypes that frequently reduce antibiotic efficacy and therapeutic success [35]. The increasing prominence of antibiofilm and anti-virulence approaches suggests a growing recognition that effective anti-MRSA interventions may require attenuating bacterial persistence and virulence rather than simple bactericidal activity alone.

The integration of computational approaches, such as molecular docking, further reflects the broader adoption of in silico prioritization and structure-guided natural-product discovery, aimed at accelerating lead identification while reducing the experimental screening burden. Similar trends have been observed across natural-product antimicrobial research, where artificial intelligence, metabolomics, and genome mining are increasingly used to identify cryptic biosynthetic pathways and prioritize pharmacologically relevant compounds [36]. Collectively, these findings suggest that research on fungi-derived anti-MRSA agents is increasingly evolving into a multidisciplinary field integrating microbiology, natural product chemistry, computational biology, and translational pharmacology.

### 4.3. Journal Ecosystem and Knowledge Dissemination

The publication landscape demonstrates that fungi-derived anti-MRSA research remains strongly anchored in journals specializing in natural product chemistry, microbiology, molecular biosciences, and antimicrobial discovery, particularly the Journal of Natural Products, Frontiers in Microbiology, Molecules, and Marine Drugs. This pattern reflects the field’s continued emphasis on fungal secondary metabolite discovery, mechanistic antimicrobial characterization, and bioactive natural-product screening rather than late-stage translational or clinical development. Similar publication patterns have been reported across the broader natural-products drug discovery ecosystem, where fungal metabolites remain important sources of structurally novel antimicrobial scaffolds against multidrug-resistant pathogens [16,36].

The strong visibility of journals such as Marine Drugs and Journal of Natural Products further highlights the importance of marine fungi, medicinal mushrooms, and endophytic fungi in anti-MRSA bioprospecting. Marine and fungal natural products possess chemically unique alkaloids, peptides, polyketides, and terpenoids with antibacterial and antibiofilm activities that continue to attract significant scientific attention [37,38,39]. Furthermore, the substantial representation of open-access journals, particularly MDPI platforms such as Molecules, Antibiotics, Marine Drugs, and the International Journal of Molecular Sciences, suggests that open-access dissemination has become an important mechanism for accelerating interdisciplinary visibility, citation accumulation, and global accessibility in antimicrobial resistance research. Rapid accessibility to antimicrobial screening methodologies, metabolomic datasets, and molecular docking workflows may facilitate broader collaborative discovery across geographically diverse research systems [40,41].

### 4.4. Fungi Remain an Underexplored Source of Anti-MRSA Compounds

Fungi are increasingly recognized as prolific producers of structurally diverse bioactive metabolites; however, they remain significantly underexplored as sources of anti-MRSA compounds compared to bacterial and plant-derived natural products [4,42]. This underutilization results primarily from biological, technological, analytical, and economic barriers that collectively impede the discovery and development of fungi-derived therapeutics targeting MRSA, rather than from an inherent lack of antimicrobial potential. As summarized in Table 3, the principal limitations associated with fungi-derived anti-MRSA compounds include dependence on specific cultivation conditions, the presence of cryptic biosynthetic pathways, metabolite complexity, limited antibacterial potency, and insufficient exploration of fungal diversity.

A significant challenge in discovering fungal anti-MRSA compounds is the complexity of fungal cultivation and metabolite expression. Many fungal species require highly specific environmental parameters, including defined nutrient compositions, pH ranges, salinity, oxygen levels, and symbiotic interactions, to achieve optimal growth and secondary metabolite production [43,44]. Unlike bacterial systems, which are readily cultured under standardized laboratory conditions, fungal growth is highly sensitive to environmental fluctuations. This sensitivity leads to poor reproducibility and inconsistent metabolite yields across studies. Slow-growing, dormant, stress-dependent, or unculturable fungal taxa are particularly difficult to isolate and maintain using conventional microbiological techniques, restricting access to potentially novel anti-MRSA metabolites. Furthermore, standard laboratory media may not induce the biosynthesis of bioactive compounds, resulting in many fungal biosynthetic pathways remaining inactive or poorly expressed.

As a result, substantial fungal metabolic diversity likely remains undetected under routine laboratory conditions. Another major limitation is the presence of cryptic or silent biosynthetic gene clusters (BGCs) within fungal genomes. Many fungi possess extensive biosynthetic machinery capable of producing diverse antimicrobial metabolites; however, these pathways frequently remain transcriptionally silent under standard cultivation conditions [45]. Consequently, the therapeutic potential of fungal metabolites against MRSA may be significantly underestimated. The inability to activate or identify these cryptic biosynthetic pathways limits the discovery of structurally novel antimicrobial compounds with unique mechanisms of action against resistant pathogens. Furthermore, the chemical and metabolic complexity of fungal secondary metabolites complicates anti-MRSA drug discovery. Fungal metabolites often exhibit highly diverse and structurally complex chemical architectures, which makes purification, dereplication, structural elucidation, and bioactivity characterization particularly challenging [46].

Historically, antimicrobial discovery research has disproportionately prioritized bacteria and medicinal plants due to their greater accessibility, simpler cultivation requirements, and more established extraction and screening methodologies [47]. In contrast, fungal bioprospecting has received comparatively less scientific attention, despite fungi possessing highly versatile secondary metabolic pathways capable of generating structurally unique antimicrobial molecules. Endophytic fungi constitute another highly promising yet insufficiently explored source of anti-MRSA metabolites [48,49]. These microorganisms inhabit internal plant tissues and frequently produce secondary metabolites that contribute to ecological defense and host protection. However, challenges related to the isolation, cultivation, and characterization of endophytic fungi continue to hinder their systematic exploration. This suggests that substantial fungal biodiversity with potential anti-MRSA activity remains undiscovered.

**Table 3:** Major Factors limiting the Discovery, Characterization, and Translational Development of Fungi-Derived Anti-MRSA Compounds.

Category	Limiting Factors	Scientific and Translational Implications	References
Biological Challenges	Cryptic or Silent Biosynthetic Pathways	Many fungi possess biosynthetic gene clusters (BGCs) that remain transcriptionally silent under standard laboratory conditions, leading to the underestimation of fungal metabolic diversity and therapeutic potential against MRSA.	[45]
	Biofilm-Mediated MRSA Resistance	MRSA biofilm formation limits antimicrobial penetration, enhances persister-cell survival, and reduces the efficacy of fungi-derived compounds despite promising *in vitro* antibacterial activity.	[7]
Chemical and Metabolic Complexity	Structural Diversity and Metabolite Complexity	Fungal secondary metabolites exhibit highly diverse and chemically complex structures, complicating purification, structural elucidation, dereplication, and functional characterization.	[46]
	Variable or Limited Antibacterial Potency	Some fungi-derived compounds demonstrate weaker antibacterial activity relative to clinically established antibiotics, limiting immediate translational applicability.	[50]
Production and Cultivation Challenges	Culture Condition Dependency	Fungal metabolite biosynthesis is highly sensitive to environmental and nutritional conditions, resulting in inconsistent antimicrobial production and reduced reproducibility across studies.	[44]
	Medium-Specific Metabolite Expression	Conventional laboratory media may fail to stimulate the biosynthesis of anti-MRSA compounds, causing potentially bioactive fungal isolates to remain undetected.	[42]
Technological and Analytical Barriers	Inefficient Identification of Biosynthetic Gene Clusters (BGCs)	Limited genomic characterization and inefficient BGC prediction hinder the discovery of novel antifungal metabolites with anti-MRSA activity.	[51]
	Dependence on Advanced Analytical Platforms	Identification and validation of fungi-derived antimicrobials often require high-cost technologies such as LC-MS/MS, molecular docking, metabolomics, and computational modeling.	[7,46]
Regulatory and Ethical Constraints	Regulatory Complexity in Drug Development	The translation of fungi-derived antimicrobials into therapeutic products is limited by complex regulatory approval pathways, standardization challenges, and safety validation requirements.	[52]
	Ethical and Bioprospecting Concerns	Ethical issues associated with biodiversity access, fungal bioprospecting, and intellectual property rights may delay research progression and commercialization.	[52]
Economic and Industrial Limitations	High Development and Scale-Up Costs	Industrial-scale production, purification, and commercialization of fungal bioactive compounds require substantial financial investment and bioprocess optimization.	[53]

### 4.5. Emerging Technologies Accelerating the Discovery of Fungi-Derived Anti-MRSA Compounds

Recent advances in computational, genomic, and biotechnological methods are significantly enhancing the discovery and development of fungi-derived anti-MRSA compounds by overcoming traditional limitations in fungal natural product research [36,54]. Conventional bioassay-guided isolation is labor-intensive, time-consuming, and often inefficient at identifying structurally novel antimicrobial metabolites. In response, computational tools such as artificial intelligence (AI), machine learning, molecular docking, and in silico pharmacokinetic prediction platforms are now used to prioritize fungal metabolites with potential anti-MRSA activity before extensive laboratory validation [55,56]. These approaches accelerate hit identification, reduce experimental costs, and improve the preliminary prediction of compound toxicity, drug-likeness, and molecular interactions with MRSA-associated targets, including penicillin-binding proteins, efflux pumps, and biofilm-related virulence factors [57,58]. As a result, computational screening is increasingly integrated into fungal natural product discovery pipelines.

Advances in genomics, metabolomics, and bioinformatics are facilitating deeper exploration of the biosynthetic potential of fungi. Through comparative genomics, transcriptomics, and metabolomic profiling, researchers are increasingly able to identify candidate antimicrobial pathways and predict fungal metabolites with potential anti-MRSA activity [59,60]. Technologies such as CRISPR-Cas genome editing, heterologous expression systems, epigenetic modification, and synthetic biology are also being explored to activate silent biosynthetic gene clusters and improve metabolite production [61]. These strategies may help address one of the longstanding challenges in fungal drug discovery, namely the limited expression of bioactive compounds under conventional cultivation conditions. In addition, high-resolution analytical platforms, including liquid chromatography–mass spectrometry (LC-MS), nuclear magnetic resonance (NMR), and molecular networking approaches, have improved the dereplication and structural characterization of fungal metabolites, thereby reducing the repeated isolation of previously identified compounds [62,63].

Biotechnological innovations are also advancing the translational development of fungi-derived anti-MRSA therapeutics by improving compound optimization, production scalability, and antimicrobial delivery strategies. Advances in fermentation engineering, co-culture systems, microfluidics, and nanotechnology-based delivery platforms are being investigated to enhance metabolite yield, stability, and bioavailability while addressing production-related challenges [16,64]. In addition, combinatorial biosynthesis and metabolic engineering approaches enable the generation of structurally modified fungal metabolites with improved antimicrobial activity and reduced toxicity [65]. Emerging platforms that integrate AI-assisted metabolite prediction with automated high-throughput screening are being explored to accelerate the identification of candidate anti-MRSA compounds from large fungal libraries [55,66]. Collectively, these multidisciplinary technologies are shifting the discovery of fungi-derived anti-MRSA agents toward a more predictive and systems-oriented research framework. Table 4 summarizes these emerging approaches.

### 4.6. Translational Gaps and Future Clinical Implications

Despite the growing conceptual maturity of the field, analyses of multidimensional scaling and conceptual structure reveal substantial translational gaps between discovery-stage investigations and clinically relevant applications. Clinical themes such as “bacteremia,” “sepsis,” and “intensive care unit” remain weakly integrated with dominant clusters centered on natural products, endophytic fungi, and antibacterial activity, indicating that much of the literature remains confined to exploratory *in vitro* screening models. This limitation reflects a broader challenge in natural-product antimicrobial research, where many compounds demonstrating promising *in vitro* activity ultimately fail in translational development due to toxicity, instability, poor pharmacokinetics, limited bioavailability, or inadequate *in vivo* efficacy [78].

In addition, considerable variability in antibiofilm assays and MRSA susceptibility testing continues to hinder reproducibility and cross-study comparability. Previous reports have similarly emphasized that many natural-product antimicrobials lack validation in clinically relevant infection models, including wound infections, systemic MRSA infections, and biofilm-associated disease models [79]. The relative separation of clinical infection themes from central discovery clusters therefore highlights the need for standardized methodologies, mechanistic validation, toxicity profiling, pharmacodynamic characterization, and rigorous animal infection studies capable of supporting translational progression.

The increasing prominence of secondary metabolites, antibiofilm activity, endophytic fungi, and molecular docking nevertheless suggests that fungi-derived antimicrobials may contribute to future adjunctive or alternative therapeutic strategies against multidrug-resistant and biofilm-associated MRSA infections. This direction is clinically relevant because MRSA biofilms remain highly refractory to conventional antibiotics and contribute substantially to chronic wounds, osteomyelitis, catheter-associated infections, and implant-related infections [80]. As summarized in Table 5, fungal-derived anti-MRSA compounds originate from diverse fungal taxa and exhibit varying degrees of antibacterial potency. However, future translational advancement will likely require stronger integration of metabolomics, genome mining, synthetic biology, nanotechnology-assisted delivery systems, and omics-guided compound prioritization to improve therapeutic precision and scalability. Future investigations should also prioritize the synergistic evaluation of fungal metabolites with existing antibiotics to determine whether fungal-derived compounds can restore antimicrobial susceptibility or reduce the emergence of resistance. Collectively, these findings suggest that while fungi-derived therapeutics remain predominantly within the preclinical discovery phase, continued methodological refinement and translational integration may strengthen their future relevance within anti-MRSA drug development frameworks.

### 4.7. Commercial Development Landscape

Although the present scientometric analysis did not directly assess patent databases, several emerging research themes demonstrate increasing translational and commercial potential within the field. The rising focus on antibiofilm activity, endophytic fungi, secondary metabolites, molecular docking, and antimicrobial resistance indicates a shift from exploratory screening toward the identification and optimization of therapeutically relevant compounds. Among these themes, antibiofilm agents are particularly promising due to the critical role of biofilm formation in MRSA persistence and treatment failure. Similarly, bioactive secondary metabolites from endophytic fungi are attractive candidates for future drug development because of their structural diversity and potential novelty. The growing use of molecular docking and computational approaches reflects efforts to accelerate lead identification and optimization, which are essential steps in the pharmaceutical development pipeline [86]. However, the limited number of toxicity studies, pharmacokinetic evaluations, and clinically relevant *in vivo* investigations indicates that most fungal-derived anti-MRSA candidates remain in the early stages of translational development. Future research that integrates patent landscape analyses, preclinical validation, and industry partnerships is necessary to more accurately assess the commercial readiness of promising fungal-derived antimicrobial compounds.

## 5. Limitations of the Study

This study has several limitations that should be considered when interpreting the findings. First, the analysis relied exclusively on the Scopus database, which may underrepresent non-indexed, regional, and non-English publications, particularly studies from low- and middle-income countries with limited visibility in indexed databases. Citation-based indicators may also favor older publications and highly collaborative research systems, potentially introducing citation and visibility bias within the dataset. Furthermore, the search strategy intentionally incorporated broad fungal and antimicrobial descriptors to maximize retrieval sensitivity and capture the diverse terminology used across fungal natural product and antimicrobial research. While this approach enhanced literature coverage, it may have initially retrieved records with indirect relevance to fungi-derived anti-MRSA compounds. Although manual screening, keyword harmonization, metadata standardization, and data-cleaning procedures were conducted to improve dataset specificity and reduce database noise, some inconsistencies in author affiliations, institutional names, and terminology and the residual inclusion of broadly related studies cannot be entirely ruled out.

In addition, scientometric analyses evaluate publication trends, thematic structures, and collaborative networks rather than the experimental quality, reproducibility, mechanistic validity, or clinical efficacy of fungi-derived antimicrobial compounds. Consequently, the findings should be interpreted as indicators of research evolution, scholarly productivity, and thematic development rather than direct measures of therapeutic effectiveness. This limitation is particularly relevant because much of the literature identified in this study remains exploratory and predominantly preclinical, with limited integration of clinically relevant infection models, pharmacokinetic evaluation, toxicity profiling, and *in vivo* validation. Moreover, the present analysis did not assess patent activity, commercial development pipelines, or regulatory advancement of fungal-derived anti-MRSA compounds. Future studies integrating multiple bibliographic databases, patent landscapes, translational outcome analyses, and clinical validation trends may provide a more comprehensive understanding of the global development and therapeutic translation of fungi-derived anti-MRSA research.

## 6. Conclusions

This scientometric study demonstrates a substantial expansion in research on fungi-derived antimicrobials targeting MRSA over the past three decades, with notable acceleration since the 2010s. This trend reflects increasing global interest in fungal natural products as alternative strategies to address antimicrobial resistance. Thematic and conceptual analyses indicate a shift from broad antimicrobial screening toward more specialized, mechanistic investigations, including studies on antibiofilm activity, molecular docking, endophytic fungi, secondary metabolites, and antimicrobial resistance. Research productivity and international collaborations are predominantly concentrated in highly resourced countries, particularly China and the United States, underscoring the need to enhance research capacity and collaborative networks in biodiversity-rich but underrepresented regions. The thematic evolution of the field suggests that future research should prioritize the translational development of promising bioactive compounds rather than continued discovery of fungal extracts. The growing emphasis on antibiofilm and molecular-based approaches demonstrates recognition that effective anti-MRSA therapies must address both antimicrobial resistance mechanisms and biofilm-associated persistence. Consequently, future investigations should focus on mechanism-of-action studies, toxicity and pharmacokinetic evaluations, standardized antimicrobial testing protocols, and validation in clinically relevant animal and biofilm infection models. Furthermore, emerging computational approaches, such as molecular docking, genome mining, artificial intelligence-assisted drug discovery, and metabolomics-guided screening, present significant opportunities to accelerate the identification and optimization of fungal-derived anti-MRSA candidates. The findings reveal a persistent disconnect between compound discovery and therapeutic translation. Although numerous fungal metabolites have demonstrated antimicrobial potential, relatively few studies have progressed to preclinical or clinical development. Bridging this gap will require multidisciplinary collaboration among microbiologists, natural product chemists, pharmacologists, bioinformaticians, and clinicians to translate fungal-derived bioactive compounds into viable therapeutic interventions. In summary, this study maps the global research landscape and identifies critical priorities to guide future efforts in developing clinically relevant fungal-derived therapeutics against MRSA and other multidrug-resistant pathogens.

## Figures and Tables

**Table 1:** Top 10 Scopus-indexed journals publishing research on fungi-derived antimicrobials against MRSA.

Rank	Journal Title	CiteScore 2024	Publisher	TP	TC	ACP	h-Index
1st	Journal of Natural Products	6.5	American Chemical Society	46	1991	43.28	24
2nd	Frontiers in Microbiology	8.5	Frontiers Media S.A.	38	970	25.53	18
3rd	Journal of Antibiotics	4.8	Springer Nature	36	753	20.92	16
4th	Molecules	8.6	Multidisciplinary Digital Publishing Institute (MDPI)	30	1565	52.17	16
5th	Natural Product Research	5.2	Taylor & Francis	26	528	20.31	15
6th	Fitoterapia	3.9	Elsevier	25	580	23.20	14
7th	Antibiotics	8.7	Multidisciplinary Digital Publishing Institute (MDPI)	24	410	17.08	11
8th	Marine Drugs	10.1	Multidisciplinary Digital Publishing Institute (MDPI)	23	815	35.43	16
9th	Chemistry and Biodiversity	3.5	John Wiley & Sons	16	237	14.81	6
10th	International Journal of Molecular Sciences	9.0	Multidisciplinary Digital Publishing Institute (MDPI)	16	455	28.44	7

Abbreviation: TP: Total publication; TC: Total citation; ACP: Average citation per paper.

**Table 2:** Top 10 institutions publishing research on fungi-derived antimicrobials against MRSA.

Rank	Affiliation	Country	TP
1st	Chinese Academy of Sciences	China	55
2nd	University of Chinese Academy of Sciences	China	29
3rd	King Saud University	Saudi Arabia	28
4th	CNRS Centre National de la Recherche Scientifique	France	27
5th	Al-Azhar University	Egypt	23
6th	Prince of Songkla University	Thailand	22
7th	Ministry of Education of the People’s Republic of China	China	22
8th	University of Mississippi	USA	20
9th	Thailand National Center for Genetic Engineering and Biotechnology	Thailand	20
10th	Institute of Microbiology Chinese Academy of Sciences	China	20

Abbreviation: TP: Total publication.

**Table 4:** Emerging Computational, Genomic, and Biotechnological Approaches Accelerating Fungi-Derived Anti-MRSA Compound Discovery.

Category	Approaches	Description	Remarks	References
Computational Approaches	Molecular Docking	Used to predict binding interactions of fungal compounds with MRSA proteins.	Isolated compounds from *Aspergillus terreus* showed stable interactions with MRSA protein active sites. Kaempferol 3-gentiobioside was identified as a potent inhibitor of MRSA-associated proteins through docking and molecular dynamics simulations.	[67]
Computational Approaches	Machine Learning (ML)	QSAR models and ML workflows used to predict antibacterial activity of fungal metabolites.	ML-based predictive models demonstrated high accuracy in screening compounds with anti-MRSA activity.	[41,68]
Computational Approaches	Protein–Protein Interaction (PPI) Networks	Applied to identify MRSA resistance-associated targets and their interactions with fungal bioactive compounds.	PPI network analysis identified key molecular targets associated with MRSA survival and resistance mechanisms.	[69]
Genomic Approaches	Whole-Genome Sequencing (WGS)	Used to identify biosynthetic gene clusters (BGCs) responsible for secondary metabolite production.	WGS analysis of *Diaporthe kyushuensis* identified 98 BGCs, approximately 60% of which were potentially novel.	[70]
Genomic Approaches	18S rRNA Gene Analysis	Applied for molecular identification and characterization of fungal strains.	*Emericellopsis minima* and *Trichoderma virens* were identified as promising fungi capable of producing anti-MRSA metabolites.	[71,72]
Biotechnological Approaches	Bioactivity-Guided Fractionation	Fractionation of fungal extracts to isolate and identify active anti-MRSA metabolites.	*Trichoderma virens* produced gliotoxin with strong anti-MRSA activity, while *Aspergillus* sp. yielded macrocyclic γ-lactams with bactericidal effects.	[72,73]
Biotechnological Approaches	OSMAC Strategy	Optimization of culture conditions to activate cryptic BGCs and enhance metabolite diversity.	OSMAC increased metabolite diversity in *Diaporthe kyushuensis* and Amazonian fungi, resulting in the production of novel bioactive compounds.	[70,74]
Biotechnological Approaches	SEM Analysis	Used to examine ultrastructural changes in MRSA cells following exposure to fungal extracts.	Fungal metabolites induced cell wall rupture, membrane disruption, and morphological abnormalities in MRSA cells.	[71,75]
Biotechnological Approaches	LC-MS/MS and GC-MS	Chemical profiling platforms for identification and characterization of fungal metabolites.	Analytical profiling identified major anti-MRSA metabolites, including diketopiperazines, gliotoxin, and integric acid.	[72,76]
Biotechnological Approaches	Co-Culturing	Used to enhance the biosynthetic capacity and metabolite production of endophytic fungi.	Co-culturing approaches reduced IC50 values of fungal extracts against MRSA and improved antimicrobial activity.	[77]

**Table 5:** Representative fungal-derived compounds with reported anti-MRSA activity and their translational status.

Fungal Source	Habitat	Representative Compound(s)	Reported Anti-MRSA Activity	Mode of Action	Cytotoxicity	References
*Alternaria alternata*	Plant-associated/Endophytic	Dibenzo-α-pyrones	MIC: 8–128 µg/mL	Exact mechanism not fully elucidated; proposed interference with bacterial growth and metabolism	Not reported	[81]
*Bionectria* sp.	Endophytic	Peptaibol, Tetramic acid derivative	Active against MRSA	Peptaibols generally disrupt bacterial membrane integrity and ion transport	Not reported	[82]
*Aspergillus tritici*	Soil-derived	Aspetritone A, Terphenyllin derivative	Activity superior to chloramphenicol	Proposed inhibition of bacterial growth; mechanism requires further elucidation	Compound 5 exhibited reduced cytotoxicity toward mammalian cells	[83]
*Xylaria longipes*	Endophytic/Wood-associated	Integric acid, Eremoxylarin C	Strong antibacterial activity	Not fully characterized	Not reported	[76]
*Talaromyces* sp.	Marine-derived	Compound 8	MIC: 0.125–0.25 µg/mL	Membrane disruption and antibiofilm activity	Low cytotoxicity reported in tested cell lines (if reported in source)	[84]
*Trichoderma virens*	Soil-derived	Gliotoxin	MIC: 25 µg/mL; MBC: 50 µg/mL	Biofilm inhibition; suppression of biofilm-associated genes; oxidative stress induction	Known mammalian cytotoxicity; requires careful therapeutic optimization	[72]
*Pestalotiopsis microspora*	Endophytic	2,4-Di-tert-butylphenol	MIC: 32 µg/mL	Membrane disruption resulting in bactericidal activity	Low to moderate cytotoxicity reported in some studies	[85]
*Emericellopsis minima*	Marine-derived	Cyclopentanol, Isothiazole, Benzoic acid derivatives	MIC: 0.8–1 mg/mL	Causes structural damage to MRSA cells	Not reported	[71]

## Data Availability

Not applicable.
